# Maladie d'Aïnhum (dactylolyse spontanée) : à propos d'un cas Clinique

**DOI:** 10.11604/pamj.2014.19.60.4839

**Published:** 2014-09-23

**Authors:** Kitembo Feruzi Maruis, Sangwa Milindi Cédrick, Kakinga Zabibu Mireille, Mutomb Jean Felix

**Affiliations:** 1 Université de Lubumbashi, République Démocratique du Congo; 2 Institut de Recherche en Science de la Santé, Antenne de Lubumbashi, République Démocratique du Congo

**Keywords:** Aïnhum, dactylolyse spontanée, Pseudoaïnhum, amputation, Ainhum, Spontaneous dactylolysis, Pseudoainhum, amputation

## Abstract

La vraie maladie d'Aïnhum est est une pathologie d’étiologie inconnue, associée à une bande de constriction autour du cinquième orteil, principalement chez les adultes de peau noire en milieu tropical. Le terme Pseudoaïnhum désigne les autres formes de constriction des doigts et orteils. Les cas de Pseudoaïnhum sont de plus en plus décrits dans la littérature. Nous présentons ici un cas de la maladie d'Aïnhum reçu au stade III de la pathologie et qui a bénéficié d'une amputation du cinquième orteil.

## Introduction

La maladie de Aïnhum, aussi appelée dactylolyse spontanée est une condition pathologique au cours de laquelle un sillon de striction tissulaire est formé autour de la partie proximale du cinquième orteil conduisant à son autoamputation [1, 2]. Elle a été plus rapportée chez les sujets noirs d'Afrique, de l'inde de l'ouest, et de l'Amérique du sud [3]. La maladie intéresse le cinquième orteil et peutse retrouver de façonbilatérale aux deux orteils [4]. Elle évolue en quatre stades à partir d'une fissuration circonférentielle à la base du cinquième orteil jusqu’à une auto amputation de celui-ci (amputation spontanée ou dactylolyse spontanée [3]. La maladie d'Aïnhum se différentie du Pseudoaïnhum par le fait que ce dernier intéresse les autres orteils (ou les doigts) excepté le cinquième. En phase précoce, le traitement médical consiste en une corticothérapie; à ce stade une plastie en Z ou une greffe de peau est possible, tandis qu'au stade évolué de la maladie, une amputation chirurgicale est incontournable [5].

## Patient et observation

**Figure 1:**
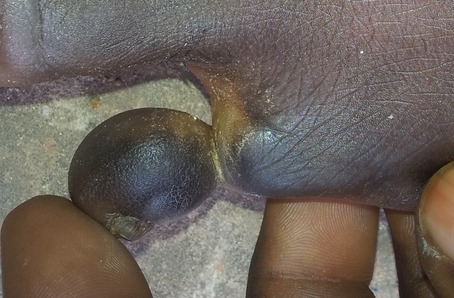
Vu de face avant amputation

**Figure 2:**
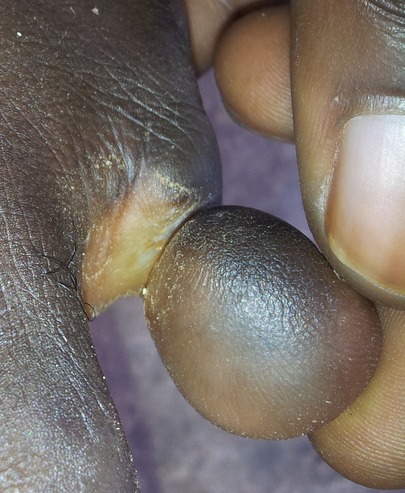
Vu de profil avant amputation

**Figure 3:**
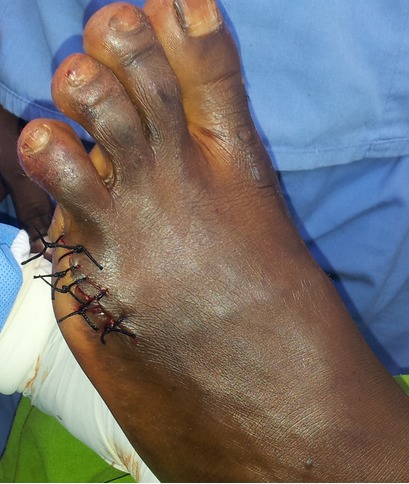
Vu de face après amputation

**Figure 4:**
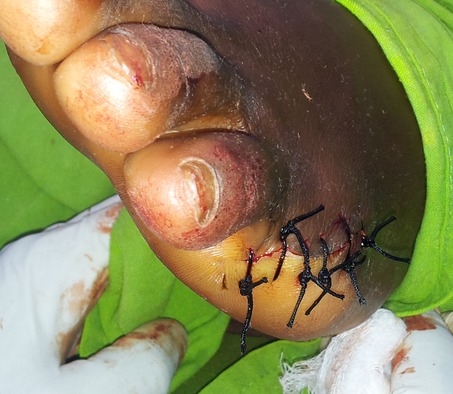
Vu de profil après amputation

Il s'agit d'un patient âgé de 27 ans de bas niveau socio-économique, qui a consulté le service de chirurgie de l'Hôpital Général de Référence Jason Sendwe de Lubumbashi pour une déformation douloureuse au niveau du cinquième orteil gauche. Le début remontait a plus de trois mois par une fissuration annulaire à la base de l'orteil qui a évolué progressivement et sans aucune consultation, ni traitement. Aucune notion de tabagisme ni même de pathologie similaire n'a été signalé chez le patient. Ses antécédents collatéraux et héréditaires étaient sans particularité. Cependant la notion de marche prolongée pied nus a retenu notre attention. A l'examen physique, nous avons noté une fissuration à la base du cinquième orteil gauche (Figure 1,Figure 2), laissant voir par endroits l'os en profondeur. L'orteil en aval était noirâtre avec une diminution de la sensibilité tactile et algésique. La partie en amont de la striction était sensible. Le reste d'examen clinique était sans particularité. Les examens biologiques étaient normaux. La radiographie du pied a noté des images d'ostéolyse à l'endroit de la striction. Une amputation du cinquième orteil a été réalisée (Figure 3, Figure 4); les suites post-opératoires étaient simples.

## Discussion

La vraie maladie d'Aïnhumest une pathologie d’étiologie inconnue, associée à une bande de constriction autour du cinquième orteil, principalement chez les adultes de peau noire en milieu tropical [2]. Neumann a utilisé le terme pseudoaïnhum pour désigner les autres formes de constriction des doigts et orteils [6]. Pendant huit ans, c'est le deuxième cas rencontré dans notre hôpital, soit une prévalence hospitalière estimée à 0,04%. Dans leur étude, Marcos et col ont pu observer que pendant 22 ans, (1977 à 1999), sur les radiographies de 6000 patients suspectés, seuls 102 (1,7%) ont présenté une dactylolyse spontanée. Dans cette série constituée uniquement des populations de peau noire et d'origine africaine, la dactylolyse spontanée n'a été associée à aucune autre pathologie [7].

La pathologie à une prédominance masculine. Elle intéresse aussi bien le côté droit que le côté gauche. Les formes bilatérales de la pathologie sont fréquemment rencontrées [4]. Notre patient a présenté une atteinte unilatérale gauche, son cas nécessite un suivi pour un dépistage précoce d'une éventuelle atteinte du cinquième orteil du côté opposé. Nous n'avons trouvé chez notre patient aucune association des pathologies qui pourrait guider vers une étiologie quelconque. L’étiologie de la maladie d'Aïnhum restée jusqu’à ce jour non élucidée. On pense qu'en plus de l’évidence sur la prédilection ethnique, les facteurs étiologiques ci-après sont associés à cette affection: le traumatisme, les infections mycosiques, la syphilis, la sclérodermie, la lèpre, les formations chéloïdiennes, les désordres des vaisseaux et nerfs périphériques, les toxines végétales, l'ostéomyélite, la filariose, la schistosomiase (schistosomaintercalatum) [8]. Chez notre patient, aucune association entre la maladie d'Aïnhum et les pathologies infectieuses tropicales n'a été retrouvée. Bien que la notion de marche pieds nus soit mentionnée, le patient ne reconnaît aucun rapport directe entre le début de sa pathologie et un traumatisme évident au niveau de l'orteil ou du pied. Selon Marcos et Browne [7, 9], la maladie d'Aïnhum est considérée comme un désordre primitif de la peau qui est observée chez les Africains noirs et leurs descendantsailleurs. Cela est dû à une tendance congénitale à une réponse fibroblastique excessive aux différents stimuli, spécialement aux traumatismes et infections chroniques. Le cas isolé d'Aïnhum chez la patiente de la race blanche a été apparenté au pseudoaïnhum apparaissant comme une manifestation des pathologies spécifiques [3].

La radiographie du patient a montré une ostéolyse au niveau de la phalange, signe pathognomonique de la maladie au stade III. Selon Marcos, cette modification osseuse est toujours constante [7]. Venu consulter au stade avancé de la maladie, la possibilité d'une conservation de la phalange (plastie ou greffe) de notre patient était impossible étant donné la section spontanée de tous les tissus mous. L'amputation est la seule possibilité chirurgicale dans le traitement tardif de l'Aïnhum [2, 5, 7].

## Conclusion

La maladie d'Aïnhum ou dactylolyse spontanée est une pathologie idiopathique affectant le cinquième orteil. Plus rencontrée chez les sujets de peau noirs vivant dans les régions chaudes, elle a une prédominance masculine. Elle est souvent bilatérale et évolue en quatre stades depuis la fissuration circonférentielle de l'orteil jusqu’à son auto-amputation. Un diagnostic et une intervention précoces préviennent les lésions irréversibles de tissus.
